# BtcA, A Class IA Type III Chaperone, Interacts with the BteA N-Terminal Domain through a Globular/Non-Globular Mechanism

**DOI:** 10.1371/journal.pone.0081557

**Published:** 2013-12-02

**Authors:** Chen Guttman, Geula Davidov, Adi Yahalom, Hadassa Shaked, Sofiya Kolusheva, Ronit Bitton, Shiran Barber-Zucker, Jordan H. Chill, Raz Zarivach

**Affiliations:** 1 Departments of Life Sciences and the National Institute for Biotechnology in the Negev (NIBN), Ben-Gurion University of the Negev, Be'er Sheva, Israel; 2 Ilse Katz Institute for Nanoscale Science and Technology, Ben-Gurion University of the Negev, Be'er Sheva, Israel; 3 Department of Chemical Engineering, Ben Ben-Gurion University of the Negev, Be'er Sheva, Israel; 4 Department of Chemistry, Bar Ilan University, Ramat Gan, Israel; University of California Merced, United States of America

## Abstract

*Bordetella pertussis*, the etiological agent of “whooping cough” disease, utilizes the type III secretion system (T3SS) to deliver a 69 kDa cytotoxic effector protein, BteA, directly into the host cells. As with other T3SS effectors, prior to its secretion BteA binds BtcA, a 13.9 kDa protein predicted to act as a T3SS class IA chaperone. While this interaction had been characterized for such effector-chaperone pairs in other pathogens, it has yet to be fully investigated in *Bordetella*. Here we provide the first biochemical proof that BtcA is indeed a class IA chaperone, responsible for the binding of BteA's N-terminal domain. We bring forth extensive evidence that BtcA binds its substrate effector through a dual-interface binding mechanism comprising of non-globular and bi-globular interactions at a moderate micromolar level binding affinity. We demonstrate that the non-globular interactions involve the first 31 N-terminal residues of BteA287 and their removal leads to destabilization of the effector-chaperone complex and lower binding affinities to BtcA. These findings represent an important first step towards a molecular understanding of BteA secretion and cell entry.

## Introduction

A large number of plant, animal and human gram negative pathogens utilize the type III secretion system (T3SS) for virulence and host immunomodulation purposes. *Bordetella pertussis*, the causative agent of the “whooping cough”, is one such pathogen in which the T3SS is essential for its virulence and persistence in the lower respiratory tract [1], [2]. The T3SS is a multi-component system composed of an injectisome apparatus, a protruding needle-like hollow superstructure anchored into the double membrane of gram negative pathogens and which serves as a means for specialized proteins (effector protein) to be secreted directly into the host cells [3]. Within the bacterial cytosol effector proteins are commonly escorted or chaperoned by non-secreted proteins termed class I chaperones. These chaperons are further sub classified to class IA and IB so to differentiate between single effector binder and multi-effector binder (also termed multi-cargo). These chaperons are typically small (14–17 kDa) acidic heart-shaped homodimers. Albeit sharing little sequence similarity, chaperons from various pathogens maintain the same structure composed of a 5-strand beta sheet framed by a two-helix bundle and one alpha-helix on either side [3]–[5]. Biochemical and structural studies revealed that chaperons bind to their cognate effector via a dedicated chaperon binding domain (CBD) positioned 20–150 residues from the effector's N-terminal end [5], [6]. Binding was shown to be mediated via a loosely sequence-conserved beta strand motif (henceforth “beta-motif”) formed at the effector CBD which interacts with a hydrophobic pocket located on each chaperon subunit such that the effector CBD is wrapped around the chaperon dimer [3]–[5]. Alternatively, the chaperon can bind one beta-motif (“non-globular” binding) and an additional binding is mediated via a distal non-conserved site on the effector surface (“globular” binding) which in some cases is relayed by other functional domains [6]–[8].

To date, only one effector-chaperon duo has been identified in *Bordetella*, BteA and BtcA [2], [9]. BteA and BtcA are located approximately 2.5 Mb from the T3SS gene locus *bsc* and their expression has been shown to be coordinated, as befits a class I chaperone-effector pair [2]. BteA, a 69 kDa protein, is a highly cytotoxic agent leading to rapid non-apoptotic cell death in various infected cells upon delivery in a T3SS-dependent manner. BteA cytotoxicity is mediated via its C-terminal domain while the N-terminal domain, characterized by an ellipsoid bi-pyramidial dumb-bell shape, is responsible for both BteA localization to lipid rafts within the cell host cytosol and its interaction with its cognate chaperone, BtcA [2], [9], [10]. To this date there is no information regarding the biochemical properties and structure of BtcA, and the manner in which it interacts with BteA. In this study we address these questions by combining homology modeling, biochemical and biophysical experiments, showing that BtcA is a dimer which interacts with the BteA N-terminal (residues 1–287, BteA287) in a specific manner via three independent interfaces with a dissociation constant in the low micromolar range. Through the use of various biochemical techniques and NMR experiments we successfully mapped the BtcA binding site upon BteA287, and show that one interface is located at the N-termini which is predicted to be non-globular in nature and responsible for the complex stabilization, while the second interface, predicted to be globular, is located at the middle of the N-terminal domain and confers the main binding strength and interface. The third interface was detected in between the non-globular and globular interaction, highlighting the extensive binding of BtcA to BteA287. We thus provide a first biochemical characterization of the BtcA:BteA complex, a key contributor to *Bordetella* toxicity.

## Materials and Methods

### Homology modeling and multiple sequence analysis of BtcA

The BtcA amino acid sequence was submitted to the CPH homology modeling server (http://www.cbs.dtu.dk/services/CPHmodels/) and a monomer model (residues 6–115) was proposed based on the SycE structure (PDB accession code 1JYA)[11]. Since SycE's template was chosen by the CPH server, we sought to manually fit the model to a slightly different class IA fold, represented by SycT's symmetry-derived dimer structure (PDB accession code 2BHO [12]). The structure quality was validated through several homology modeling servers [13]–[15]. The dimer model of BtcA was then manually adjusted for minimizing clashes and subjected to minimization function via Swiss PDB viewer version 4.0.4 [16]. Structural superposition for calculating the root mean square distance (RMSD) between structures was performed by the SwissPDB viewer alternate domain fitting function. Images of BtcA, SycE and SycT models were visualized using PyMOL [17]. Multiple sequence alignments (MSA) of BtcA and BteA287 were performed using Jalview with the Muscle algorithm [18], [19].

### Cloning, expression and purification of BtcA

The cloning of BtcA into pET28(+) was described previously [2]. The ligated plasmid was transformed into E. coli BL21 Codon+ competent bacteria cells after which selected colonies were grown to mid-exponential phase. At this point expression of the proteins was induced by addition of isopropyl β-D-1-thiogalactopyranoside (IPTG) to 1 mM final concentration for 18 hr at 25°C. Cells were collected by centrifugation at 6000 rpm for 7 min at 4°C after which the pellet was resuspended with binding buffer (20 mM imidazole, 300 mM NaCl, 20 mM Tris pH 8, 0.02% Triton X-100). The cells were lysed by French Press (Thermo Scientific, Asheville, NC) and centrifuged at 45,000 rpm for 45 min at 4°C. Batch purification was conducted by applying the supernatant to buffer equilibrated Ni-NTA beads (Novagen), followed by 3 washing steps using Econo-Column (Bio-Rad, Hercules, CA): buffer 1 (50 ml of 300 mM NaCl, 20 mM Tris pH 8, 20 mM imidazole), buffer 2 (50 ml of 600 mM NaCl, 20 mM Tris, pH 8, 30 mM imidazole) and buffer 3 (50 ml of 300 mM NaCl, 20 mM Tris pH 8, 40 mM imidazole). Elution was performed in the presence of elution buffer (350 mM NaCl, 20 mM Tris pH 8, 300 mM imidazole) after which eluted samples of BtcA were concentrated and injected onto a Superdex 75 26/60 column (GE healthcare, Little Chalfont, UK) pre-equilibrated with BtcA buffer (20 mM Tris pH 8, 350 mM NaCl). Selected peaks were fractionated, pooled and concentrated to 12 mg/ml.

### MALDI-TOF/MS analysis

Matrix was prepared by dissolving sinapinic acid (Sigma-Aldrich, Rehovot, Israel) in TA (33% Acetonitrile, 0.1% TFA) to saturation. The protein samples were mixed with the matrix at 10∶1 and 100∶1 v/v matrix∶sample ratios. Each mixture (1 µl) was dispensed on the MALDI target plate and dried at ambient temperature. Samples were analyzed on a Reflex IV (Bruker Daltonics, Bremen, Germany) MALDI-TOF mass spectrometer using 337 nm radiation from a nitrogen laser. The spectra of BtcA and BtcA-BteA287 complex were recorded in linear mode within a mass range from m/z 1,000 to 22,000 and 20,000 to 150,000, respectively.

### Circular dichroism analysis

Circular dichroism measurements were conducted with a J750 Spectropolarimeter (JascoInc, Mary's Court, Easton, USA). A BtcA sample was prediluted to 0.2 mg/ml in buffer containing 50 mM NaCl, 20 mM Tris pH 8 and measured with a 0.1 cm optical path Suprasil quartz cuevette (Hellma GMBH & Co., Müllheim, Germany). Spectra profiles of the samples were measured at a wavelength range of 200–260 nm at ambient temperature with bandwidth set to 1 nm, scan speed set to 10 nm⋅min^−1^ and a time constant of 4 seconds. Secondary structure content was predicted through the use of K2D algorithm via Didchroweb online server [20], [21].

### Analytical size exclusion chromatography, molecular weight determination and model fitting

Purified BtcA (29 mg/ml) was loaded onto a Suprdex 75 10/300 (GE Healthcare, Little Chalfont, UK) equilibrated with 20 mM Tris buffer pH 8, 350 mM NaCl, 20 mM EDTA and elution volume was monitored via absorbance at 280 nm. A calibration curve was generated by plotting the elution volume of a protein standard kit (GE healthcare, Little Chalfont, UK) against their known molecular weight. The elution volume of BtcA was used to extract the molecular weight from the established curve.

### Cross linking assays

For crosslinking assays a 3 mM solution of ethylene glycol bis[succinimidylsuccinate] (EGS, Thermo Scientific Pierce) was serially diluted 1∶1 with PBS. Protein samples (BtcA and BtcA-BteA287 complex) were diluted to approximately 4 mg/ml in PBS and were incubated with the different EGS concentrations for 30 minutes at RT. The reactions were quenched by addition of 1 M Tris pH 8, followed by buffer exchange to 10 m M Tris 8, 5 mM NaCl (for MALDI-TOF analysis) or supplemented with sample buffer (for SDS-PAGE analysis).

### Characterization of the BtcA-BteA287 interaction by protection from trypsinization

BteA287 and BtcA were mixed at ∼1∶3 molar ratio (2 mg/ml and 3 mg/ml, respectively) and incubated at 37°C for 30 and 60 minutes. Samples containing the complex and either the chaperon or effector were subjected to limited proteolysis by addition of 1∶6000 Trypsin (Sigma-Aldrich, Israel). Reaction was carried out at RT for 30 and 60 minutes after which proteolysis was quenched by addition of sample buffer. The samples were resolved via 17.5% SDS-polyacrylamide gel and stained by Coomassie Blue stain. Newly-appearing bands were identified by the Edman degradation assay (Biological Services department, Weizmann Institute of Science, Rehovot, Israel).

### Determining the BtcA stoichiometry by SEC-RALS

Purified BtcA (525 µg) were loaded onto Superdex 200 column (10/300, GE Healthcare) connected to a triple detector array (TDA) model 305 (Viscotek Ltd., Houston, TX) which consisted of a static light scattering cell with a photodiode dectector at 90° for right angle light scattering (RALS), a deflection refractometer (Ri), as well as a photometer. The column was equilibrated with buffer (20 mM Tris pH 8.0, 350 mM NaCl, 20 mM EDTA). All data were acquired using the Omnisec software (Viscotek Ltd., Houston, TX). Bovine serum albumin (Sigma) was used for TDA internal constants calibration. The incremental refractive index, dn/dc, was set to 0.185. The RALS data in combination with the concentration as determined with the deflection refractometer provided an estimation of the molecular mass.

### Microscale thermophoresis (MST)

The proteins BteA287 and BteA32-287 were labeled by using the protein labeling kit BLUE-NHS (NT-495 blue fluorescent dye) according to the manufacturer's instructions. BtcA was brought to 200 and 100 µM concentration (BteA287 and BteA32-287, respectively) and was serially 1∶1 diluted in 20 mM Tris 8, 350 mM NaCl, 20 mM EDTA and 0.05% Tween-20. Labeled BteA287 and BteA32-287 were mixed at 1∶1 (v/v) and incubated for 10 minutes at RT. Samples were loaded on hydrophilic silicon capillaries (K004 Monolith™) and MST measurements were performed during 30 seconds on a Monolith NT.115 (NanoTemper Technologies, Munich, Germany) at 20°C (blue LED power set to 50% and infrared laser power set to 40% power). Data of 3 independent measurements were averaged, analyzed and fitted in Origin 8 software (OriginLab, Guangzhou, P.R. China) using the logistic dose-response algorithm.

### SAXS data collection

Prior to performing the SAXS experiments all protein samples were subjected to SEC purification to eliminate products of complex formation or aggregation. BtcA, BtcA∶BteA287 and BtcA∶BteA32-287 samples were diluted each with 20 mMTris 8, 350 mM NaCl, 20 mM Tris 8, 300 mM NaCl and 10 mM Tris 8, 300 mMNaCl, respectively. SAXS measurements were performed at the French national synchrotron facility SOLEIL, on the SWING beamline. The incident beam energy was 12 keV. The sample to detector (Aviex CCD) distance was set to 1892 mm, covering a q-range of 0.004–0.7 Å^−1^. All experiments were temperature controlled at 25°C. Typically 55 successive frames of 0.5 s each were recorded for both protein solution and its corresponding buffer. Each frame was first angularly averaged and the final spectrum and experimental error were obtained by averaging over all frames and subtracting the pure solvent spectrum from the sample spectrum. Intensities were scaled using the scattering of water [22].

### SAXS data analysis and envelope model

The radius of gyration (r_g_) was evaluated using the Guinier approximation [23]. The GNOM program was used to obtain the Pair-distance distribution functions, the corresponding maximum dimension of protein complexes (Dmax)and to determine the value for r_g_ from the entire scattering profile [24]. *Ab initio* envelopes were generated by the program DAMMIN (Svergun, 1999) using atomic radii set to the dummy atom packing radius determined by DAMMIN without imposing symmetry operation [24]. Several DAMMIN runs were performed for every sample and an averaged dummy ball model (DBM) was generated by DAMAVER [25]. The generated DBMs were manually fitted on BtcA homology model via the Coot software [26] and visualized by PyMOL [27].

### NMR spectroscopy

NMR samples of BteA287 were prepared as previously described [10]. In order to study the binding mode of BtcA to BteA, a 0.4 mM sample of uniformly labeled ^2^H, ^13^C, ^15^N-BteA287 in 20 mM phosphate buffer (pH 7.5), 100 mM NaCl and 7% ^2^H_2_O was placed in a Wilmad NMR tube (Wilmad Labglass, Vineland, NJ, USA). Experiments were conducted at a static magnetic field of 16.4 T and using a Bruker DRX700 spectrometer equipped with a cryogenic TCI probe and z-gradients. Assignment of BteA287 backbone resonances was performed using a suite of TROSY-based triple resonance experiments, including the HNCO and HN(CA)CO, HN(CO)CA and HNCA, and HN(CO)CACB and HNCACB [28], [29]. Each pair of spectra provided intra- and inter-residual connectivities which together allowed the assignment of a system of peaks to a particular ^1^H-^15^N moiety along the BteA287 sequence. To follow the interaction between BteA287 and BtcA a series of 2D-^1^H,^15^N-TROSY-HSQC (tr-HSQC) spectra were recorded for the sample before addition of BtcA and at five different BtcA concentrations corresponding to BteA∶BtcA ratios of 1∶0.25, 1∶0.47, 1∶0.73, 1∶1 and 1∶1.43, with BtcA molar ratios referring to the dimeric protein. A tr-HNCO spectrum was recorded for the final sample to facilitate the identification of peaks. Each spectrum was acquired for 30 minutes at 303 K. BtcA was added from a 25–30 mg/ml stock in 20 mM Tris buffer (pH 8.0) and 350 mM NaCl, leading to final concentrations of 0.18 and 0.26 mM of BteA and BtcA, respectively. Addition of similar amounts of equivalent buffer had a negligible effect on the spectrum. Spectra were processed using the TopSpin 2.1 package (Bruker BioSpin, Karlsruhe, Germany).

## Results

### Bioinformatics analysis suggests BtcA is a class IA chaperone

**Figure 1:**
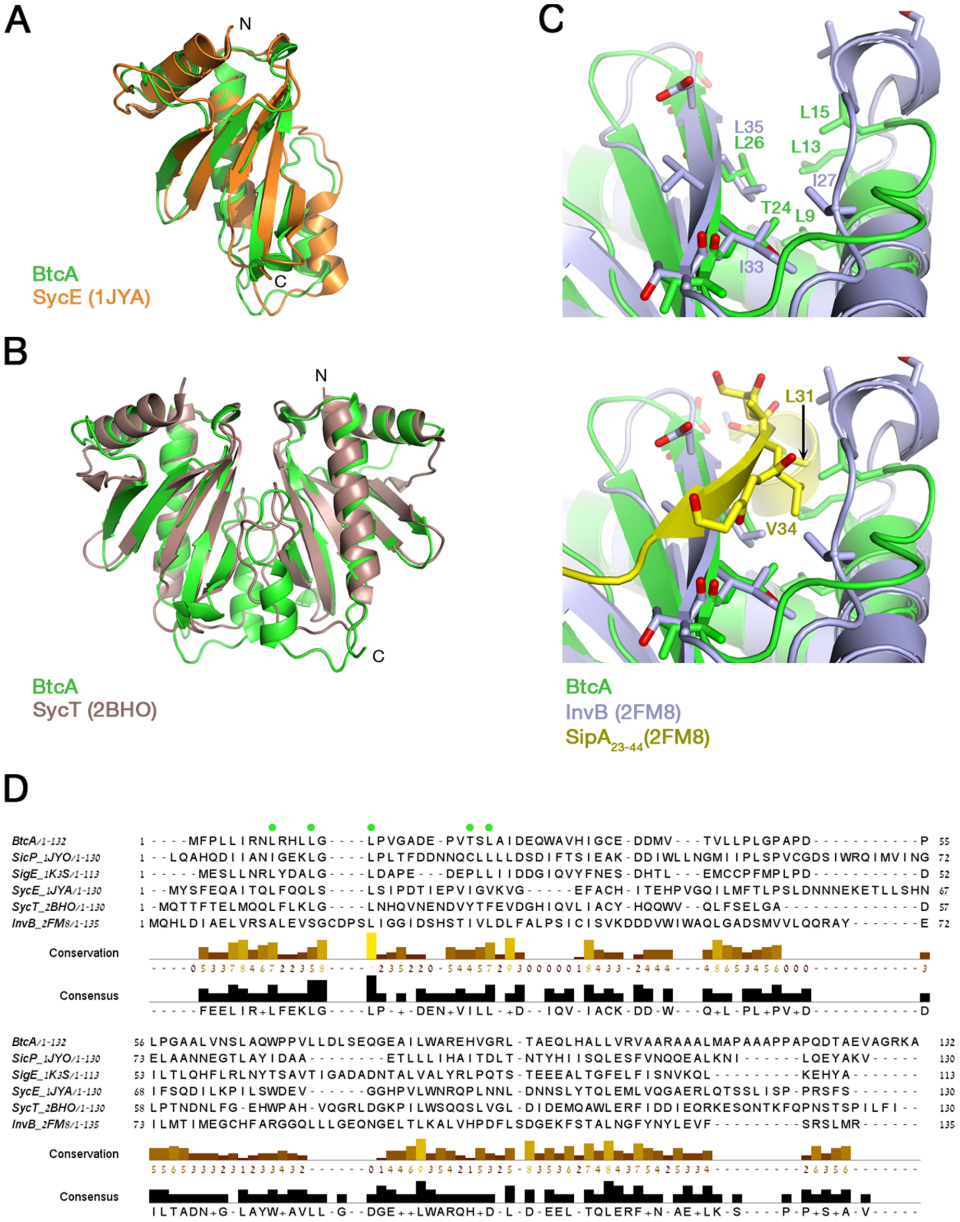
Comparison of BtcA model and sequence to known class I chaperons. Superposition of monomer (A) and dimer (B) BtcA homology models over the determined structures of SycE (1JYA, [11]) and SycT (2BHO, [12]), respectively. (C) Superposition of BtcA model over the determined InvB:SipA complex, focusing on the β-pocket secondary structure, highlighting key residues with and without the presence of yellow-colored SipA's β-motif (lower and upper panel, respectively). (D) MSA of BtcA and other class I chaperons demonstrating conservation among key residues that form the β-pocket motif (marked by green dots).

One of the hallmarks of the T3SS class IA chaperones is their structure of a five stranded β-sheet flanked by three α-helices on either side [3], [4], [30]. We speculated that if BtcA is a class IA chaperone its structure will be similar to other class IA chaperons. For this purpose, we generated a homology model through the use of CPH modeling server [31]. The modeling server chose SycE's deposited structure as a template and generated an initial monomeric model of BtcA (Figure 1A and Materials and Methods). The generated model received a high score of reliability (10.9) and exhibited an excellent fit to the structure of SycE (RMSD 1.17 Å), demonstrating the typical α/β mix structure of class IA chaperones. Since T3SS chaperones are typically associated as homodimers [3], [5], [30], we modeled a BtcA dimer on to the determined structure of SycT (Figure 1B) [12] with good results (RMSD 1.65 Å), further supporting our notion that BtcA is a class IA chaperon.

We extended our bioinformatic analysis to the dimeric form of BtcA to examine its homology to another chaperone-effector complex. We superimposed the BtcA model on SipA-InvB complex structure and demonstrated that the BtcA model indeed has a conserved β-strand binding pocket, or “β-pocket” described by Lilic et al [6] (Figure 1C, top panel; RMSD 1.75 Å). Furthermore, we were able to show that the β-pocket could enable the fitting of the N-terminal fragment of SipA (residues 23–44, PDB code 2FM8). The multiple sequence alignment of several class IA chaperons highlights the conservation of several key residues, mostly leucines, which could be traced back to the BtcA model (Figure 1D). We conclude that according to bioinformatics and homology modeling BtcA is a T3SS class IA chaperon characterized by two β-pockets in its dimer form.

### BtcA is a soluble protein characterized by a prominent α/β fold

**Figure 2:**
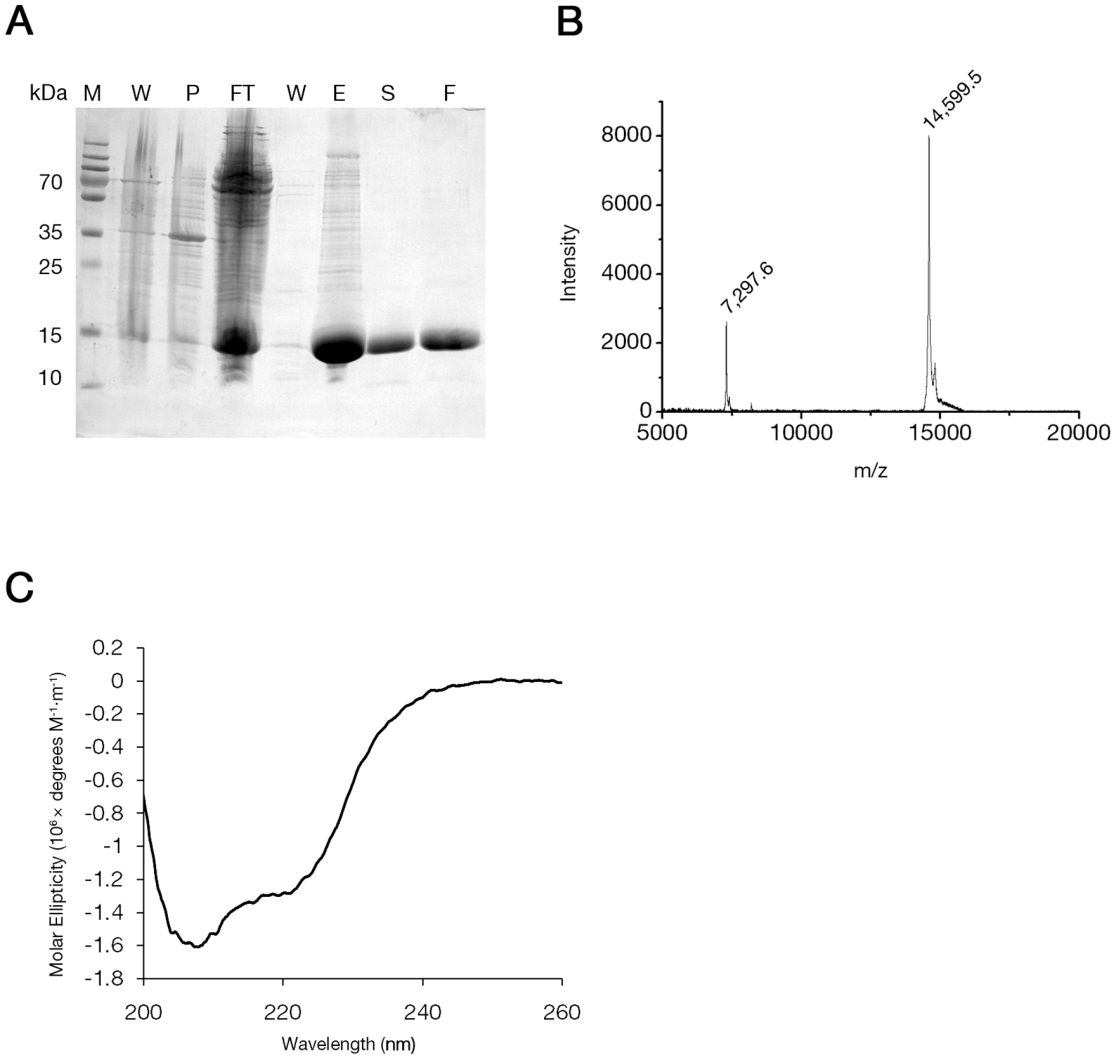
BtcA purification and fold characterization. (A) SDS-PAGE analysis of BtcA Ni^2+^-affinity chromatography (AC) and size exclusion chromatography (SEC). Lanes are labeled as follows: M, marker; W, whole cell lysate, P, pellet; FT, flow through; E, elution from AC column; S, elution from SEC column; F, concentrated BtcA sample. (B) MALDI/TOF-MS analysis of purified BtcA with corresponding masses (in Daltons) indicated above main peaks. (C) Circular dichroism curve for BtcA measured in 50 mM NaCl, 20 mM Tris pH 8 buffer conditions at 25°C.

For the purpose of biochemical characterization and verification of our BtcA homology model we expressed and purified BtcA using Ni^2+^-affinity chromatography and size exclusion chromatography. A large scale expression and purification has generated large quantities of BtcA at over 95% purity (Figure 2A). Matrix-assisted laser desorption/ionization time-of-flight mass spectrometry analysis (MALDI/TOF-MS) produced a major peak which corresponds to the mass of a monomer (14.5 kDa with His_6_ tag) further verifying the purity of the protein and the validity of the protein purification scheme (Figure 2B). The circular dichroism (CD) curve of BtcA at room temperature (Figure 2C, black line) exhibited a double minimum at 208 nm and 222 nm. Analysis of this curve suggests contributions of both α-helical (37%) and β-strand (26%) conformations. These figures are similar to the values obtained from the model of BtcA, in which 28 and 33 residues of the total 111 chaperone-modelled residues adopt α-helical and β-strand conformations, respectively.

### BtcA is a homodimer

**Figure 3:**
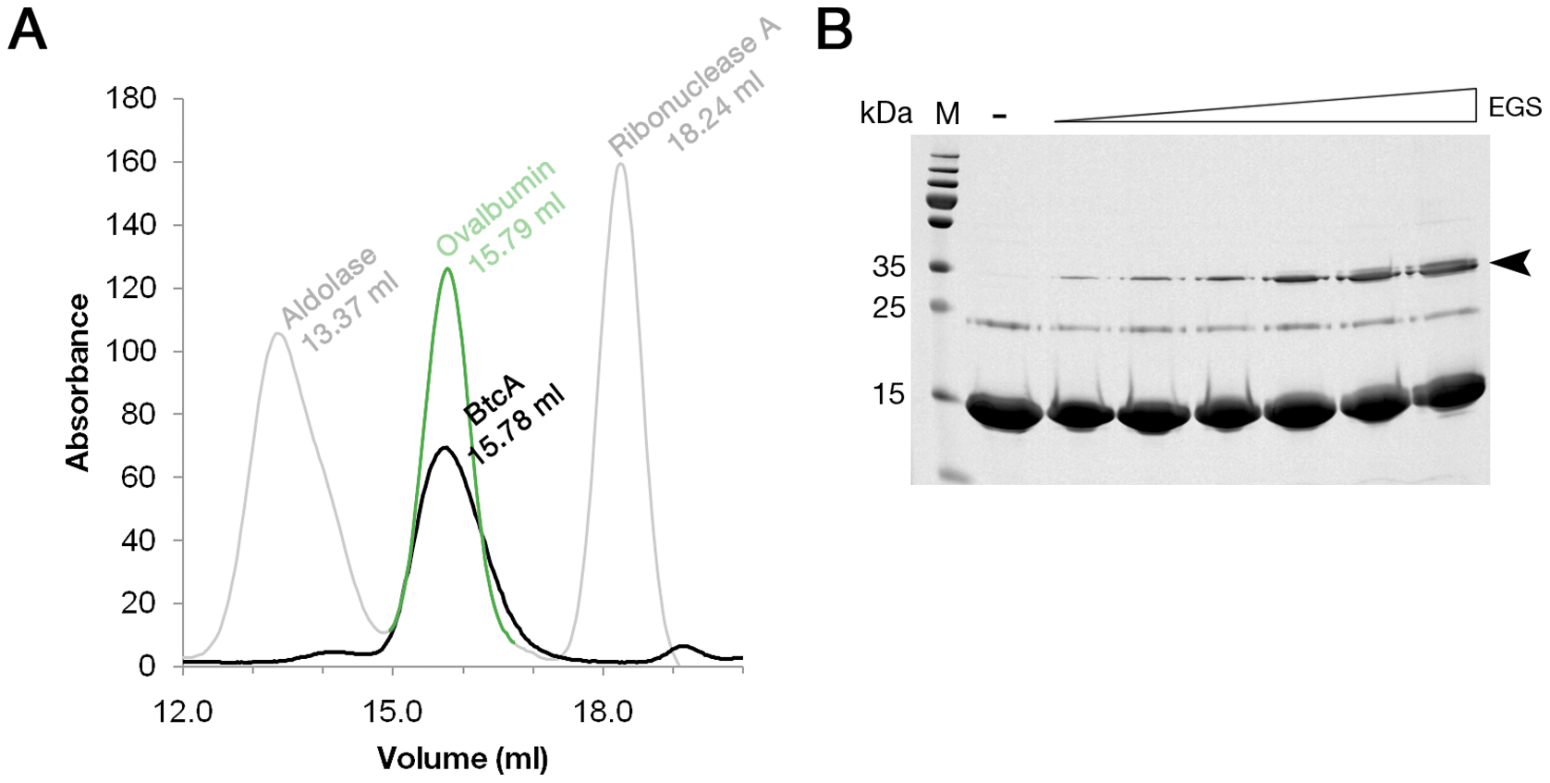
Size and oligomeric characterization of BtcA. (A) SEC elution profiles of BtcA (black), aldolase, ribonuclease (grey) and ovalbumin (green) on a Superdex 200 10-300 column. (B) SDS-PAGE analysis of BtcA crosslinked with increasing amounts of EGS. Arrow marks the expected band size of BtcA's dimer population.

Since class IA chaperons readily form homodimers we analyzed BtcA's oligomeric tendency through biochemical and biophysical approaches. The size exclusion chromatogram (SEC) of BtcA shows it to elute earlier than expected for a BtcA dimer, exhibiting an elution volume (15.79 ml) highly similar to that of the 44 kDa ovalbumin (15.78 ml) (Figure 3A, black line). Although this would suggest trimeric stoichiometry for BtcA, elution in gel filtration columns is strongly influenced by size and, to a lesser extent, by non-specific interactions with the matrix. To determine the size of BtcA more accurately we performed a cross-linking experiment on purified BtcA followed by SDS-PAGE analysis (Figure 3B). The gel exhibits an EGS dose-response pattern of BtcA shifting onto a covalently formed SDS-resistant dimer (marked by black arrow). Notably, this is the highest molecular weight species observed in the experiment. Furthermore, SEC-coupled right angle light scattering (RALS) analysis of BtcA yielded a calculated average molecular weight of ∼32 kDa, supporting our notion that BtcA is a homodimer (Figure S1). The observed variation of the calculated molecular weight across BtcA's peak is possibly due to a high-MW contaminant peak eluting just before BtcA. In summary, we conclude that BtcA forms a native homodimer, suggested by the SEC results to be slightly extended in shape.

### BtcA binds BteA's N-terminal at intermediate binding affinity

**Figure 4:**
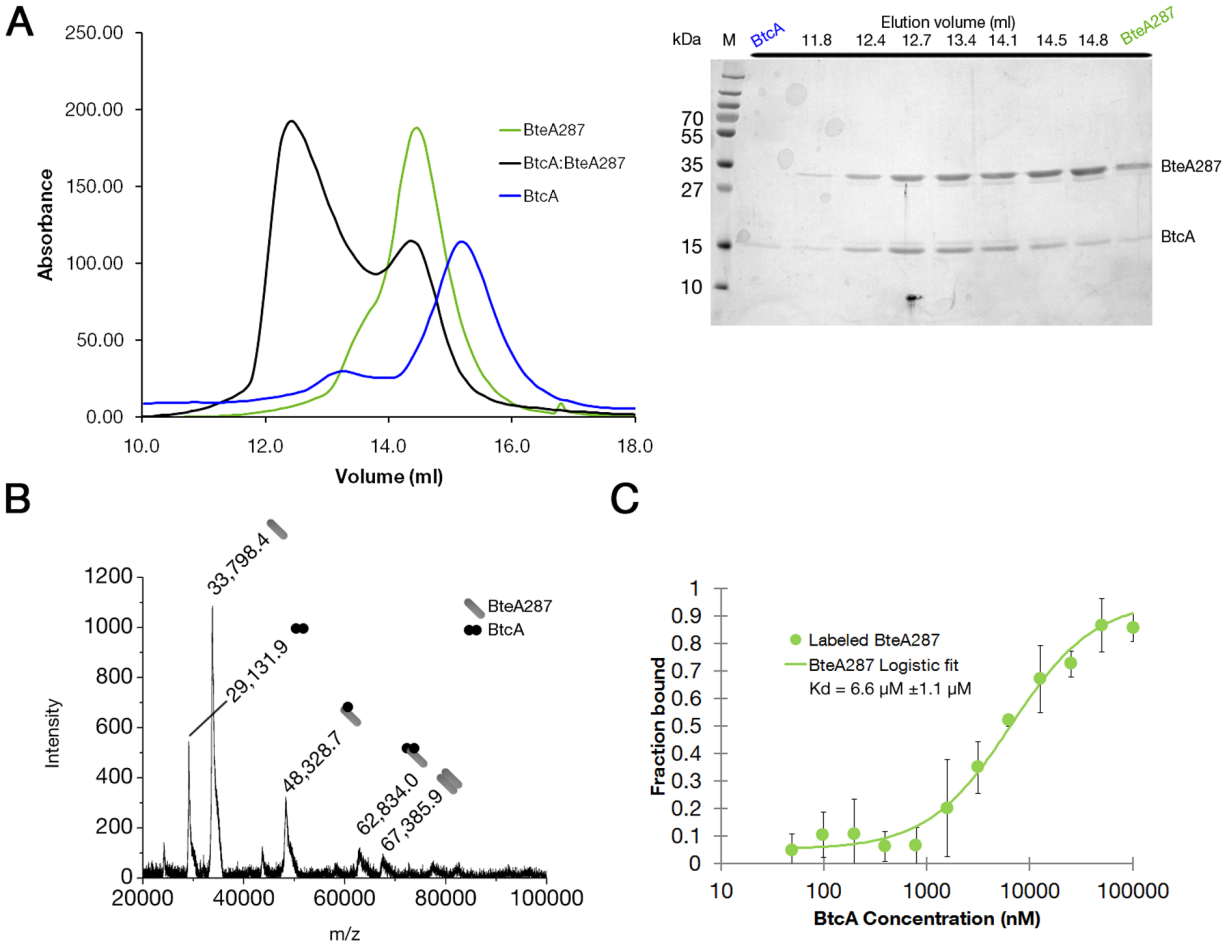
Determination of BtcA∶BteA287 complex stoichiometry and binding affinity. (A) SEC elution profiles of BteA287 (green), BtcA (blue) and BtcA∶BteA28 complex (black), and SDS-PAGE analysis of fractions collected at various elution volumes. (B) MALDI-TOF analysis of the BtcA∶BteA287 complex. Cartoons represent monomer BteA287 (grey rod), BtcA monomer (black circle), dimer BteA287 (twinned rods) and dimer BtcA (twinned circles). (C) Labeled BteA287 (green boxes) was mixed with serially diluted BtcA samples and the thermophoretic behavior was monitored at RT. Values were normalized to the fraction of bound receptor and are average of three independent experimental repeats. Values were fitted with logistic function and the denoted Kd values were extracted from the center of the curve.

Previous publications have demonstrated that BtcA interacts with BteA through the latter's first 130 residues (chaperone binding domain or CBD) via a yet-to-be determined mechanism and at unknown molecular stoichiometry. We first evaluated the affinity and stochiometry of binding of recombinant BteA287 to BtcA by analytical size exclusion chromatography (Figure 4A). Addition of BtcA to BteA samples induced a pronounced shift in the elution profile of the latter, indicating the formation of a higher molecular weight species (green curve versus black curve), identified by SDS-PAGE to contain both BteA287 and BtcA. MALDI-TOF analysis of this complex verified our observation that BtcA binds to BteA287 (Figure 4B), showing evidence of 1∶1 and 1∶2 BteA287∶BtcA ratios, as well as some dimeric BteA287.

At this stage we employed microscale thermophoresis (MST) to quantify the strength of BtcA's binding to BteA287 (Figure 4C). We have mixed labeled BteA287 with decreasing concentrations of BtcA and the measured thermophoresis was fitted with a sigmoid curve (R^2^∼0.98), extrapolating the dissociation value as 6.6 µM±1.1. We thus conclude that BtcA binds to BteA's N-terminal with medium strength and a stoichiometry of 2∶1 and 1∶1.

### BtcA binds BteA287 via two chaperon binding sites

**Figure 5:**
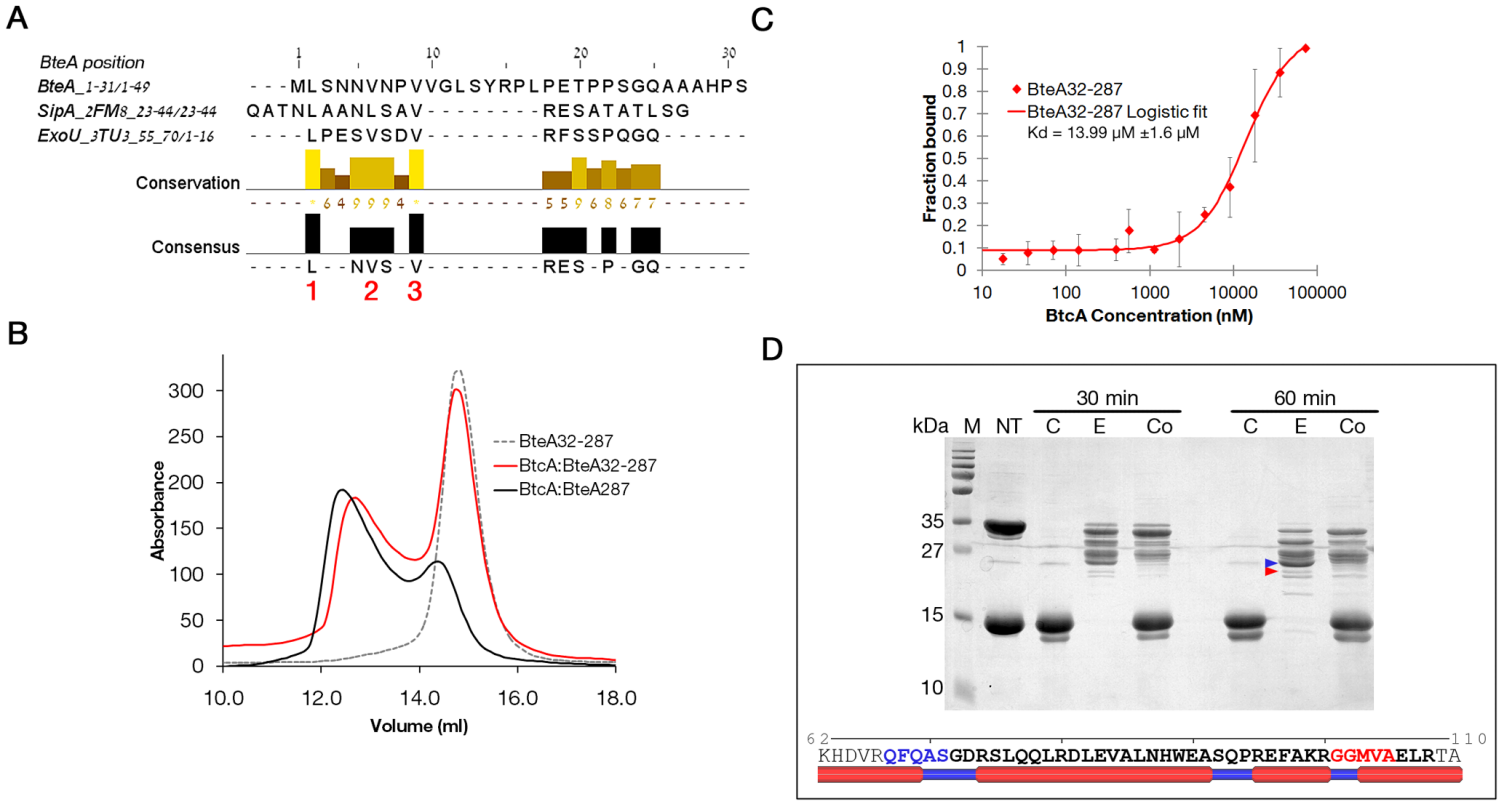
BteA contains two chaperon binding domains. (A) Sequence alignment of BteA's first 31 residues and the structurally-determined β-motif of SipA [6] and ExoU [7]. Red numbers mark key residues of the β-motif as previously suggested. (B) SEC elution profiles of BteA32-287 (hatched grey line), BtcA∶BteA32-287 (red) and BtcA∶BteA287 (black). (C) Labeled BteA32-287 (red boxes) was mixed with serially diluted BtcA samples and the thermophoretic behavior was monitored at RT. Values were normalized to the fraction of bound receptor and are average of three independent experimental repeats. Values were fitted with logistic function and the denoted Kd values were extracted from the center of the curve.

Having established the formation of the BtcA∶BteA287 complex, we proceeded to explore the mechanisms by which BtcA binds its effector. Previous publications have demonstrated that chaperones bind their respective effectors through a beta motif located at the effector's N-terminal domain [6], [32]. We postulated that the first 31 residues of BteA might harbor such a β-motif especially since our homology model indicated that BtcA might exhibit two β-motif binding pockets (Figure 1C). The multiple sequence alignment (MSA) between the first 130 residues of BteA287 (the minimal domain shown to bind BtcA) and the beta motifs residues of SipA and ExoU exhibits strong conservation of leucine and valine residues within the determined beta motif residues of SpcU and ExoU (Figure 5A). Furthermore, the general motif identified by Lilic et al [6], [L/M/I/F]XXX[L,V]XX[V,L,I,Q,H], is not found elsewhere in the CBD of BteA. Thus we hypothesized that removal of the first 31 residues will modulate binding of BtcA to BteA287. For this purpose we analyzed a complex between BteA32-287, an effector construct lacking the first 31 residues, and BtcA by analytical SEC and compared it to the BteA287∶BtcA complex elution profile at similar chaperone∶effector molar ratios (Figure 5B). Strikingly, even in the presence of BtcA over half of the truncated effector is found in the free state, suggesting the shorter BteA binds BtcA with lower affinity, in agreement with findings for complex of InvB with either SipA1-270 or SipA32-264 [6]. Further supporting this conclusion is the finding that the MST-derived dissociation values afforded a two-fold and statistically significant reduction in affinity associated with the removal of the first 31 N-terminal residues, from 6.6 µM to 14 µM (R^2^∼0.98, Figure 5C). Thus, there is compelling evidence that these 31 residues, containing the putative β-motif, play a role in binding of effector and chaperon.

Formation of a complex between BtcA and truncated BteA287 clearly indicates that an additional binding interface is involved in their interaction. This was confirmed by subjecting BteA287, BtcA and the formed complex to a time-limited trypsinization assay in which the proteolytic products were separated via SDS-PAGE (Figure 5D). We identified a 26 kDa fragment observed in the effector lane yet absent in the complex lane, suggesting a digestion site which is masked by the chaperon-effector interface. This site was mapped to the segment spanning residues 67-108 (Figure 5D) which is within the previously determined CBD region. Furthermore, we have found that this fragment is expected to be on the surface of the protein since most of the residues were predicted to be solvent-exposed by the Weighted Ensemble Solvent Accessibility (WESA) meta-predictor algorithm [33] (data not shown). We conclude that BtcA binds BteA287 via two interaction elements, a predicted-globular interaction involving the CBD region and a predicted non-globular interacting domain involving the 31 N-terminal residues of the effector.

### NMR titration experiments map the globular interacting domain

Nuclear magnetic resonance (NMR) is a well-established method for studying protein-protein interactions on the molecular level [34], [35]. As part of our ongoing effort to determine the solution structure of BteA287 we expressed and purified ^2^H, ^13^C, ^15^N-labeled labeled BteA287 (BteA287^DCN^) and acquired TROSY-based triple-resonance experiments [28], [29] for purposes of resonance assignment. The helical nature of BteA287, resulting in poor dispersion in its tr-HSQC spectrum, together with its aggregation tendencies [10] which limit sample concentration, make BteA287 a challenging target for assignment. Nevertheless, data coming from the ongoing assignment process sheds light on complex formation between BtcA and BteA287, and, importantly, pinpoints the BteA interaction surfaces.

**Figure 6:**
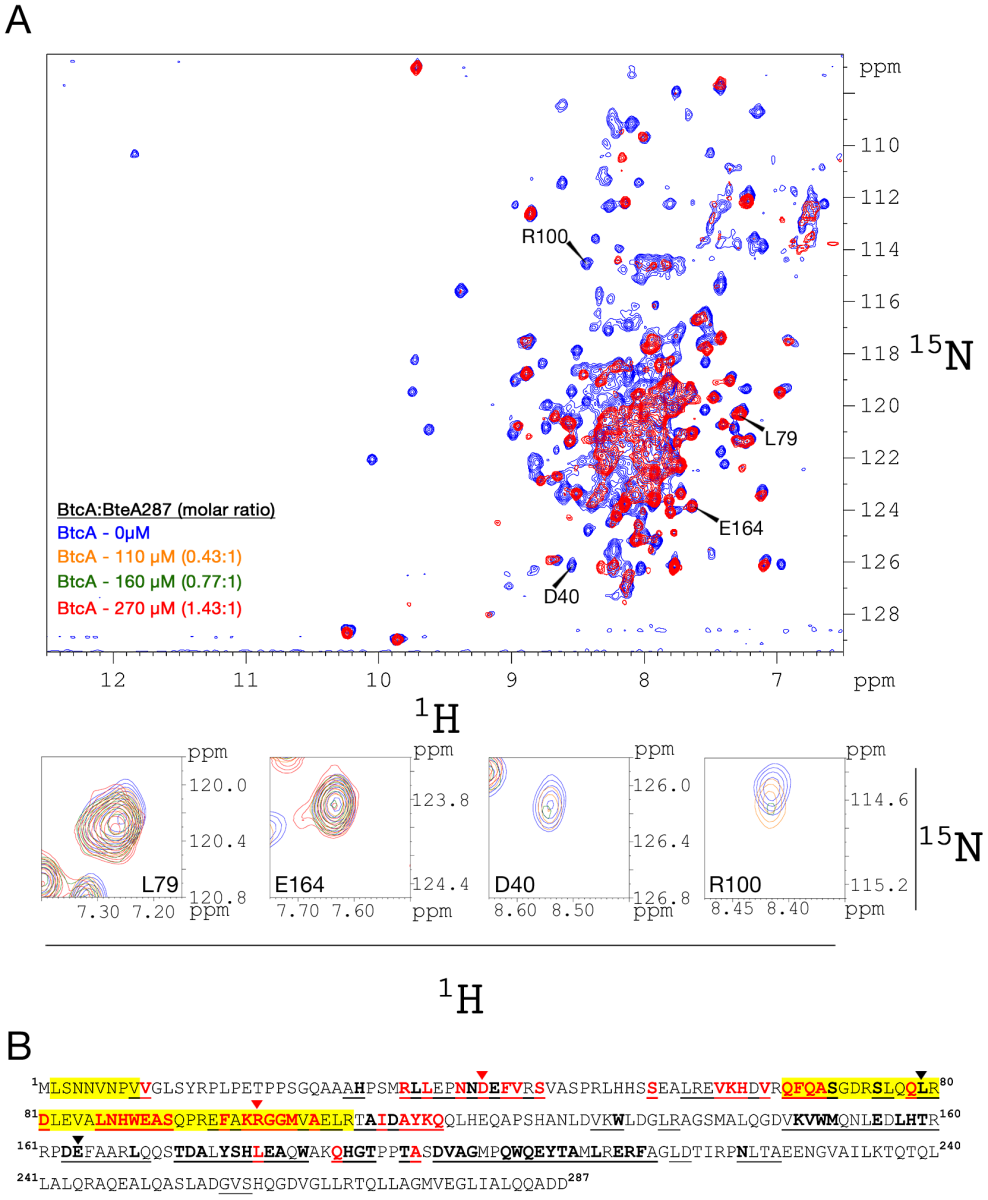
NMR-monitored titration reveals key BteA287 residues involved in the binding of BtcA. (A) Superposition of the tr-^1^H-^15^N HSQC spectra of a BteA287^DCN^ sample in the absence of BtcA (blue) and in the presence of increasing BtcA concentrations, 110 µM (orange), 160 µM (green) and 270 µM (red). Below are detailed views of four cross-peaks marked by arrow heads. (B) The BteA287 amino acid sequence showing assigned backbone resonances (underline), affected (bold red) and unaffected residues (bold black). Arrowheads correspond to peaks shown in detail. Highlighted in yellow are the currently known non-globular (residues 2–10) and globular (residues 67–108) interaction domains.

We titrated unlabeled BtcA into a BteA287^DCN^ sample (molar ratios of up to 1.43∶1 dimeric-BtcA∶BteA287) and followed changes in the effector tr-HSQC spectrum, focusing on outlying peaks which could be accurately monitored in the two-dimensional experiment (Figure 6A). Binding of BtcA results in widespread intensity loss of BteA287 peaks, suggesting that the two proteins form a complex with intermediate (micromolar) affinity characterized by an extensive interaction surface. The titration distinguishes between peaks whose intensity was independent of BtcA concentration (“unaffected peaks”), and those whose intensity is diminished as BtcA is titrated in (“affected peaks”, Figure 6A, zoomed regions), the latter representing residues, whose chemical shift is affected by binding, and thus are likely located in the binding site or its close vicinity. Mapping these two groups along the BteA287 sequence reveals a clear pattern. Of the 40 affected peaks, 29 represent residues clustered within the BteA 61-115 segment, and an additional seven emanate from residues clustered within the BteA 33-45 segment (Figure 6B). Whereas the former overlaps with the aforementioned putative globular site, the latter embodies a previously unknown interaction site between the two proteins. Based upon its predicted structure, this region of BteA is expected to be partially unstructured (residues 33–40) and partially helical (residues 41–45) as was seen previously for other effector-chaperon complexes (data not shown) [36]–[38]. In contrast, of the 50 unaffected peaks, 40 were located in the region spanning residues 150–215, suggesting it does not interact with the chaperon. Assignment levels in other regions, including the N-terminal tail of BteA, were insufficient for unambiguous determination of an interaction with BtcA. Nevertheless, the NMR titration experiment highlights the extensive binding interface between the two proteins and verifies the interaction with the predicted globular domain, and also identifies an additional BteA segment participating in chaperon binding.

### SAXS analysis of BtcA alone and in complex with BteA287 and BteA32-287

**Figure 7:**
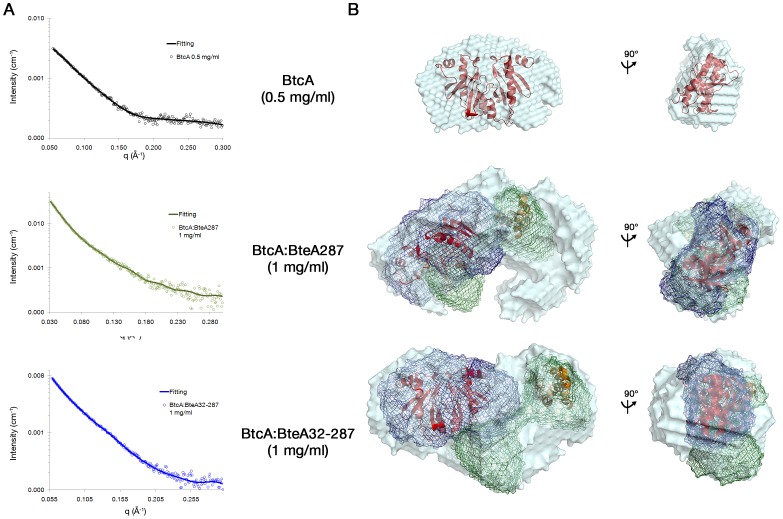
SAXS analysis of free and complexed BtcA. (A) Experimental data (circles) and the corresponding back-fitted dummy-ball model (DBM). (B) Dummy-ball envelope models (light azure) with superimposed models of BtcA (red cartoon) and BteA287 (orange cartoon, 2JPF). The DBMs of BtcA and BteA287 (blue and green mesh, respectively) have been superimposed over the DBMs of the BtcA∶BteA287 and BtcA∶BteA32-287 complexes.

In order to confirm our suggested model for BtcA (Figure 1) and its binding mode to the two forms of BteA, we collected and analyzed small-angle X-ray scattering (SAXS) data for BtcA in isolation and in complex with the two BteA constructs. The SAXS curves of free BtcA is, as expected, quite distinct from those of the complexes (Figure 7A, top panel), and the 31 N-terminal residues appear to have little effect on BteA molecular shape, in agreement with the similar R_g_ values of the two complexes (Table S1 and Figures S2 and S3).

The above-mentioned SAXS data served as a platform to generate dummy-ball models (DBMs) of BtcA alone or in complex with BteA's N-terminal variant proteins through the use of DAMMIN [39] (figure 7B, top panel). BtcA adopts a heart-like shape which is consistent with the SAXS data, and could also be successfully superimposed on the homology model of BtcA in its dimer form, further supporting our claim that BtcA is a homodimer. The two complex envelopes models exhibit similar shape and sizes and were successfully back-fitted to the SAXS data (figure 7A and B, middle and bottom panels). The generated BtcA envelope (figure 7A) as well as BteA287 envelope model superposed with the core NMR structure of BteA's N-terminal (2JPF, unpublished and trimmed as described elsewhere [10]) were fitted within the envelope of both complex envelope and found to be consistent with the binding stoichiometry and robustness of the BtcA∶BteA complex.

## Discussion

One of the hallmarks of the T3SS is the binding of effector proteins by the T3SS chaperons prior to the effector's secretion into the host cell cytosol, highlighting the importance of this interaction [3]–[5]. In this study we characterized BtcA and its interaction with its cognate effector's N-terminal domain (BteA287), currently the only chaperon-effector duo identified in the *Bordetella* genus. BtcA amino acid sequence shares little homology to other structurally-determined class IA T3SS chaperons, emphasizing the importance of structural information obtained in this study. Despite this, a model of BtcA derived from its limited similarity to other chaperons was consistent with experimental SAXS and CD data, predicting for BtcA a slightly extended homodimeric structure with a typical α/β fold (Figure 1). This structure has seen for other class IA T3SS chaperons [6], [11], [12], [32], [40].

Previously it was shown that BtcA can bind BteA via a minimal domain comprising of the first 130 residues of BteA (CBD) yet the location of the binding site and its molecular basis were left undetermined. Here we have confirmed the interaction of BtcA with BteA287 via a plethora of biochemical and biophysical approaches and determined the binding stoichiometry to be 2∶1 (chaperon∶effector), as was seen for other chaperon∶effector complexes [6], [36], [40], [41], as well as 1∶1 (as shown by MALDI-TOF). However, we find the BtcA∶BteA287 complex to differ from their counterparts in other T3SS systems in its micromolar affinity, which is 2–3 orders of magnitude weaker than previously-published chaperon∶effector determined affinities [7]. This result was obtained from MST data and confirmed by the NMR titration results. The micromolar affinity of this complex implies that the release of BteA287 from BtcA is more favorable in comparison to the above-mentioned chaperon∶effector complexes.

The mechanism through which chaperons bind to and stabilize their cognate effectors is of much interest in the field of T3SS and until now was not known for the *Bordetella* secretion system. Chaperons bind their cognate effectors via two possible mechanisms, one involving binding of the effector's two β-motif (“non-globular interaction”) to the dimeric chaperon and the other involving the combined binding of one non-globular interaction and one globular interaction. The latter is mediated in many cases via a domain which has a dedicated function within the host cell [6]. To determine which mechanism governs the interaction of BtcA with BteA's N-terminal domain, we utilized complementary bioinformatics and empirical approaches. Our study identified two possible β-pockets for BtcA, as observed for other class IA chaperons, yet only one possible β-motif peptide was identified at the N-terminal region of BteA which is within the secretion signal peptide [2], [9], [10]. Since T3SS effector-chaperon complexes involves at least two interaction sites, we speculated that at least one additional site should be located C-terminal to the β-motif yet within the CBD boundaries. For this purpose we utilized a protection assay comparing BtcA∶BteA287 complex to each of the components alone, enabling the identification of the second interaction site located at CBD's midsection (residues 67–108). While the suggested N-termini β-motif is predicted to be mostly unfolded, similar to other T3SS effector's determined β-motif, the second motif of BteA287 is predicted to be folded as interchanging alpha helices and short loops, which are characteristic of a globular domain [6]–[8]. Furthermore, NMR titration experiments interpreted in the context of our ongoing backbone assignment identified key residues located at the BtcA binding interface. Most notable was finding an additional binding surface including residues 33–45 located between the N-terminal signal peptide and the CBD. Precedents for this additional binding region, which highlights the extensive nature of the chaperon∶effector interface [6], [7], [36]–[38], [40], [42], have been previously observed for a number of effector-chaperon complexes [36]–[38]. In other BteA regions data was insufficient to unequivocally determine their involvement in binding, although it strongly suggests a contribution of the N-terminal signal peptide to the observed affinity (data not shown). We anticipate that completion of the BteA287 assignment will yield a much-needed global fold for the effector and serve as a foundation for determining its structure. However, even at this stage the NMR data draw a compelling picture as to the nature of the chaperon∶effector complex. Line shape behavior is highly consistent with an intermediate-affinity complex, and affected outlying peaks in the two-dimensional spectra were clustered in the binding regions in a statistically significant manner.

In light of this NMR-based mapping of the binding site and the possible role of the first 50 effector residues it is interesting to reconsider the binding affinities found for wild-type and truncated BteA. The SEC-RALS and MST experiments demonstrate, qualitatively and quantitatively, respectively, that while a weaker complex was formed by the truncated BteA32-287 variant, the predicted globular domain is the main mediator and driving force leading to the interaction between BtcA to BteA287. Although the difference in calculated dissociation constant suggested by MST (∼40%) may not fully account for the observed chromatogram shift, this discrepancy may originate from the intermediate affinity of the complex and inherent methodological differences. The absence of the first 31 N-terminal residues obviously obviates their contribution to binding, but also may perturb the fold of BteA residues 33-45 and affect their ability to interact with the chaperon. Further studies will be necessary to further illuminate this aspect of complex formation.

Further indication of the intimate nature of the BtcA∶BteA complex was offered by the SAXS data, allowing a comparison of R_g_ values for free and complexed BteA. The effector R_g_ increased from 3.1 [10] to 4.6 nm upon binding of BtcA, even though the chaperon R_g_ was estimated at 2.9 nm. This relatively small increase in size is consistent with our suggested model of an extensive interaction surface between the two proteins. The similar R_g_ values observed for the two BteA N-terminal variants in complex with BtcA suggests that the flexible N-terminal tail contributes little to the envelope shape and size of both complexes. This might be due to the different binding position the two variants adopt once they are bound by BtcA.

In conclusion, we present here an in-depth structural and biochemical characterization of effector-chaperon in *Bordetella*, contributing and strengthening to the currently accepted dogma of a binding site which combines interactions with predicted globular and non-globular regions, as was suggested by Lilic et al [6]. Besides providing first direct information for the only known chaperon∶effector system in *Bordetella*, this study also lays the groundwork for further and more detailed structural studies of this intriguing protein-protein complex.

## Supporting Information

Figure S1 **SEC-RALS analysis of BtcA.** Solid and dotted lines represent absorbance at 280 nm and right angle light scattering (RALS)-determined molecular weight, respectively.
(TIF)

Figure S2 **Guinier plots of SAXS data.**
(TIF)

Figure S3 **Overlap of SAXS data of BteA287 and BteA32-287 complex with BtcA.** SAXS data of BtcA in complex with either BteA287 (green) or BteA32-287 (blue) demonstrate the differences in scattering patterns.
(TIF)

Table S1 Summary of SAXS geometric data.
(DOCX)
