# Supplementary figures and images for: BtcA, A Class IA Type III Chaperone, Interacts with the BteA N-Terminal Domain through a Globular/Non-Globular Mechanism
Source: PLoS One. 2013 Dec 2;8(12):e81557. doi: 10.1371/journal.pone.0081557 (PMC3846842; doi:10.1371/journal.pone.0081557)

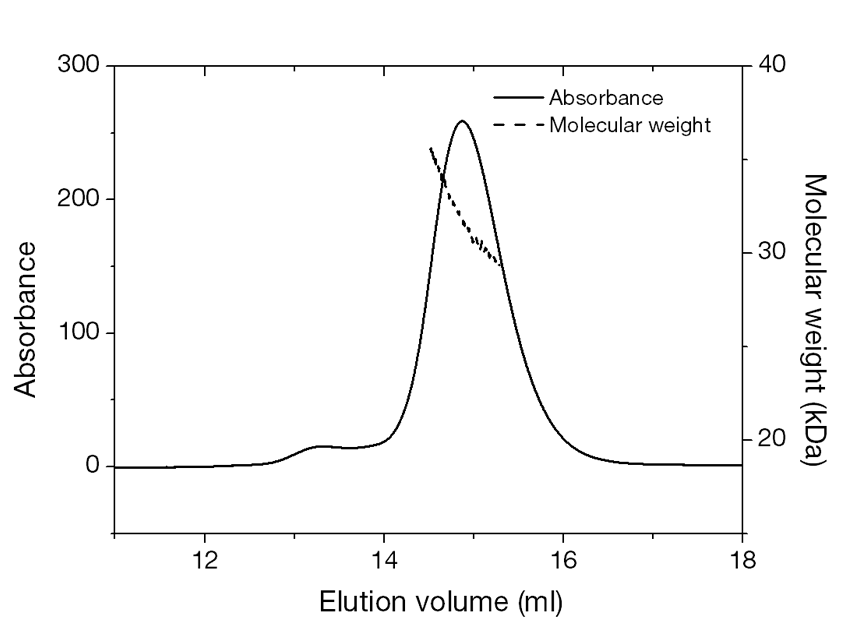

Supplement: Figure S1 — SEC-RALS analysis of BtcA. Solid and dotted lines represent absorbance at 280 nm and right angle light scattering (RALS)-determined molecular weight, respectively. (TIF) [file pone.0081557.s001.tif]

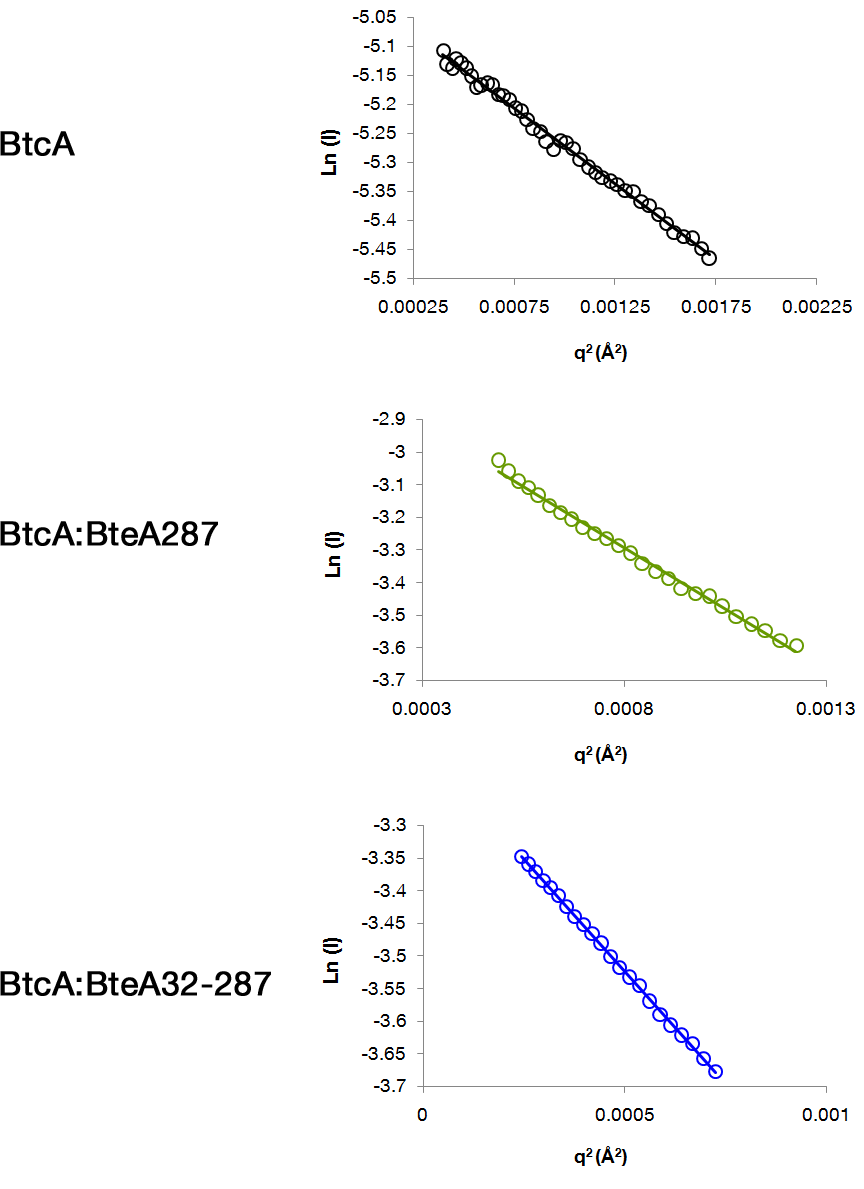

Supplement: Figure S2 — Guinier plots of SAXS data. (TIF) [file pone.0081557.s002.tif]

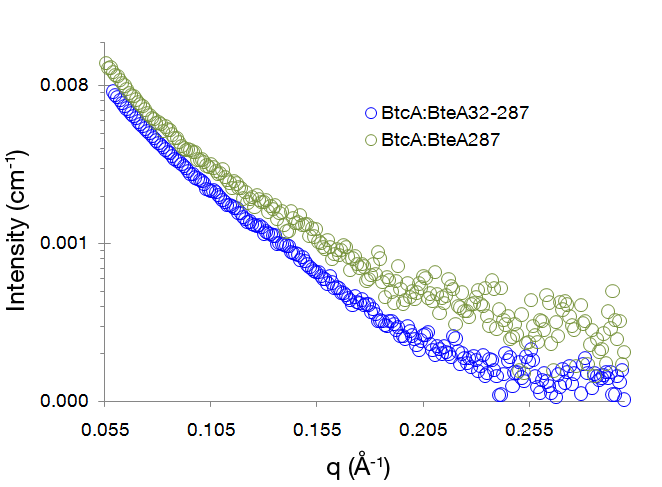

Supplement: Figure S3 — Overlap of SAXS data of BteA287 and BteA32-287 complex with BtcA. SAXS data of BtcA in complex with either BteA287 (green) or BteA32-287 (blue) demonstrate the differences in scattering patterns. (TIF) [file pone.0081557.s003.tif]
