# Supplementary material for: BtcA, A Class IA Type III Chaperone, Interacts with the BteA N-Terminal Domain through a Globular/Non-Globular Mechanism
Source: PLoS One. 2013 Dec 2;8(12):e81557. doi: 10.1371/journal.pone.0081557 (PMC3846842; doi:10.1371/journal.pone.0081557)
Supplement: Table S1 — Summary of SAXS geometric data. (DOCX) [file pone.0081557.s004.docx]

**Supporting information**

**Table S1.** Summary of SAXS geometric data

|  | Rg (Å) | | |
| --- | --- | --- | --- |
|  | **0.5 mg/ml** | **1 mg/ml** | **2 mg/ml** |
| **BtcA** | 28.05±0.06 | 29.72 ±0.16 | NA |
| **BteA287** | 43.49±0.18 | 46.2±0.03 | NA |
| **BteA32-287** | NA | 45.42±0.04 | 49.7±0.44 |
|  |  |  |  |
| NA – Not available | | | |
